# The Effect of Poly(Vinyl Chloride) Powder Addition on the Thermomechanical Properties of Epoxy Composites Reinforced with Basalt Fiber

**DOI:** 10.3390/ma13163611

**Published:** 2020-08-15

**Authors:** Danuta Matykiewicz, Kamila Sałasińska, Mateusz Barczewski

**Affiliations:** 1 Institute of Materials Technology, Faculty of Mechanical Engineering, Poznan University of Technology, Piotrowo 3, 61-138 Poznań, Poland; mateusz.barczewski@put.poznan.pl; 2 Central Institute for Labour Protection—National Research Institute, Department of Chemical, Biological and Aerosol Hazards, Czerniakowska 16, 00-701 Warsaw, Poland; kamila.salasinska@ciop.pl

**Keywords:** epoxy, poly(vinyl chloride), basalt fiber, thermomechanical properties

## Abstract

The aim of the article was to determine the effect of the poly(vinyl chloride) additive (PVC) on the thermomechanical and fire properties of epoxy composites reinforced with basalt fabric. Ten-layered composites with 2.5, 5 and 10 wt.% of PVC powder were fabricated using hand lay-up. The following features were evaluated for composites: structure (by scanning electron microscopy, SEM), thermomechanical properties (by dynamical thermomechanical analysis, DMTA), mechanical properties (in bending, tensile and interlaminar shear strength tests), hardness (using the Barcol method), thermal stability (by thermogravimetry, TGA) and fire behavior (using a cone calorimeter). It was found that the introduction of micron PVC powder into the epoxy matrix improved the thermomechanical properties of composites, such as storage module, and mechanical properties, such as flexural strength and modulus, as well as hardness.

## 1. Introduction

Technological development in various branches of industry creates the need for new construction materials [1,2,3]. Therefore, in recent years polymer composites have become a widely used construction material in many industries and the requirements for these materials are continually growing [4,5,6,7]. Due to the low strength and fire resistance of epoxy composites, various types of fillers and reinforcing fibers are introduced in order to improve their properties [8,9,10]. Due to the variety of interactions that may occur between individual components in a composite, the assessment of its properties is critical in order to determine its application potential. Epoxy resins reinforced with different types of particles can be classified as hybrid materials, which are designed using substances with different properties and form in order to achieve a significant improvement in the properties of the polymer matrix [11,12].

The following techniques are used to produce layered composites, depending on the shape, type of resin, permeability of the fibers and the time needed to make the element; hand lay-up process, resin transfer molding, vacuum-assisted resin transfer molding, and hot or cold pressing. The influence of selected forming methods on the properties of finished products has been widely described in the literature [13,14,15,16]. Basalt fiber (BF) is characterized by high strength, as well as chemical and thermal stability, which is why it has found wide application as a reinforcing material in polymer composites. Sarasini et al. proved that hybrid composites based on aramid and basalt woven fabrics have a better impact energy absorption capability than composites reinforced only with aramid fabric and enhanced damage tolerance [17]. Wang et al. described the degradation of the tensile properties of prestressed basalt fiber-reinforced polymer (BFRP) and hybrid FRP tendons in a marine environment [18]. Tirillò et al. described the effects of basalt fiber hybridization on high-velocity impact response of carbon fabric-reinforced epoxy composites [19].

Double modification of epoxy resin—through simultaneous reinforcement with a fiber filler and introducing dispersed powder filler—creates an opportunity to improve the mechanical, thermal, electrical or tribological of the composites. Li et al. used nanoparticles, such as montmorillonite, nano SiO_2_ and montmorillonite/nanoSiO_2_ double nanoparticles, for the modification of the epoxy/basalt fiber composites [20]. The combined nano-modifier composed of montmorillonite and nano SiO_2_ improved the interlaminar shear strength and impact strength of the epoxy/basalt fiber composites. Subagia et al. investigated the effect of different tourmaline micro/nano particle loading on the tensile and flexural properties of a basalt fiber-reinforced epoxy composite laminate [21]. They found that the addition of tourmaline significantly improved the tensile and flexural strength and modulus of the basalt/epoxy composite. Bulut proved that the addition of graphene nanopellets at low concentration into the epoxy matrix improves the mechanical properties of epoxy composites containing basalt fiber [22]. Natural graphite flakes used as a filler in the production of an epoxy composite reinforced with basalt fibers improved their mechanical properties, as described in the paper [23]. Khosravi et al. described the influence of the addition of multi-walled carbon nanotubes (MWCNT) to the epoxy matrix on the mechanical properties of unidirectional basalt fiber/epoxy composites [24]. While Kim et al. used MWCNT-coated basalt fibers as a reinforcement for epoxy resin [25], these composites showed highly improved flexural behaviors compared to the reference material. Abdi et al. reported that, in the case of basalt–epoxy composites modified with nano CaCO_3_, the tensile and flexural strength values increase with the amount of a introduced nanofiller as a result of improved load transfer between the nanocomposite matrix and BF [26]. In turn, Surana et al. used sawdust from the wood industry as a filler for basalt–epoxy composites, which improved the mechanical properties [27]. Obtaining a specific modification efficiency depends on the amount of polymer matrix filler used; for nanofillers it is usually up to 1% and it can reach up to 40% by weight in the case of micro fillers.

Products made of PVC are characterized by favorable properties and a relatively long life cycle. Still, finding an area to use recyclate from post-consumer PVC products seems to be an important ecological and technological issue. To the best of the authors’ knowledge, the available literature does not mention the use of powdered PVC as an effective modifier of the properties of basalt–epoxy composites. It should also be emphasized that the application of poly(vinyl chloride) powder to modify the properties of other fiber reinforced composites has not been described. In our previous work, we presented poly(vinyl chloride) powder as an inexpensive flame-retardant epoxy resin [28]. Therefore, the purpose of this work was to determine the possibility of using powdered PVC as a modifier in an epoxy composite reinforced with basalt fiber. The following features were evaluated for composites: structure (by scanning electron microscopy, SEM), thermomechanical properties (by dynamical thermomechanical analysis, DMTA), mechanical properties (in bending and interlaminar shear strength tests), hardness (using the Barcol method), thermal stability (by thermogravimetry, TGA) and fire behavior (using a cone calorimeter).

## 2. Materials and Methods

### 2.1. Materials

Epoxy resin Epidian 6 (EP6) based on bisphenol A (BPA) (viscosity at 25 °C; 10,000–15,000 mPas) with curing agent—triethylenetetramine (Z1)—both produced by CIECH Sarzyna S.A., Poland—were used as the matrix of the composites. Suspension poly(vinyl chloride) Polanvil S-67 (PVC) in the form of powder with particle size below 250 µm and basalt fiber woven fabric (BASALTEX)-type BAS 210.1270.P with 210 g/m^2^ were used as the filler. The epoxy resin was mixed with Z1 at the ratio: 13 parts Z1 per 100 parts EP6 by weight.

### 2.2. Sample Preparation

The epoxy resin was mixed using a mechanical stirrer Disperlux (proLAB, Gdańsk, Poland), with 2.5, 5 and 10 wt.% of PVC powder to the total weight of composition, respectively, under subatmospheric pressure conditions (0.2 Bar). The resulting compositions were mixed with a curing agent Z1 [28]. The epoxy composites were fabricated in a mold using hand lay-up, cured at ambient temperature (23 °C) for 24 h and post-cured at 80 °C for 3 h. They contained 10 layers of woven fabric. The samples were designated as 0PVC; 2.5PVC; 5PVC; 10PVC adequately to the PVC powder content.

### 2.3. Methods

The morphologies of the composites were monitored by Scanning Electron Microscopy (SEM). The fracture surfaces of the samples after impact tests were photographed at the magnification of 1000 with a scanning electron microscope Carl Zeiss Evo 40 (Oberkochen, Germany) and with an electron accelerating voltage of 12 kV. All materials were covered with a layer of gold.

Dynamic thermomechanical analysis (DMTA) was used to assess the thermomechanical properties of the composites using the Anton Paar MCR 301 apparatus (Graz, Austria) in the torsion mode, at a frequency of 1 Hz, in the temperature range of 25 °C–180° C and a heating rate of 2 °C/min. The glass transition temperature (Tg) was determined for the maximum tan *δ* value.

The material properties for bending were determined during a three-point bending test in accordance with DIN EN ISO 14125 using a Zwick Roell Z010 (Ulm, Germany) machine at a test speed of 1 mm/min and with a 10 kN load sensor. The width of the sample was 10 mm, the length of the span was calculated as 16-times the thickness of the sample.

Tensile mechanical behaviors were analyzed in accordance with ISO 527-4 by an INSTRON 4481 universal testing machine at 25 °C with a testing speed of 5 mm/min and a load cell of 50 kN.

Interlaminar Shear Strength Test (ILSS) was carried out in accordance with the EN2563.Zwick Roell Z010 machine at a test speed of 1 mm/min and with a 10 kN load sensor. Specimen dimensions for the short-beam shear test were 20 mm × 10 mm × 2 mm and the length of the span was 10 mm. The internal laminar shear strength of the composites was calculated using Equation (1).

(1) $$\tau =\frac{3P}{4\mathrm{bh}}$$

where: τ—the maximum ILSS at failure; P—the maximum load on the specimen; b—the width of the specimen; h—thickness of the specimen.

The hardness of the materials was determined with an ASTM D2583 standard Barcol hardness tester GYZJ-934-1 (Barber Colman Co., Loves Park, IL, USA).

The assessment of thermal stability of composites by the thermogravimetric method (TGA) was conducted in an air atmosphere in the temperature range from 30 °C to 900 °C and at the heating rate of 10 °C/min using a Netzsch TG 209 F1 apparatus (Selb, Germany) in ceramic pans. The initial decomposition temperature T10% was defined as the temperature at which the weight loss was 10% and the residual mass was determined at 900 °C. The maximum temperature of the thermal degradation and rate was also determined based on derivative thermogravimetric curves (DTG).

The fire behavior was determined by a cone calorimeter (Fire Testing Technology Ltd., East Grinstead, UK), in accordance with ISO 5660-1. The samples (100 × 100 × 4 mm^3^) were wrapped in an aluminum foil and placed on the holder in a steel frame, providing a surface area of 88.4 cm^2^. All specimens were irradiated horizontally at a heat flux of 35 kW/m2. After tests, the residues were photographed using a digital camera (EOS 400 D, Canon Inc. Tokyo, Japan).

## 3. Results

### 3.1. Composites Structure

**Figure 1:**
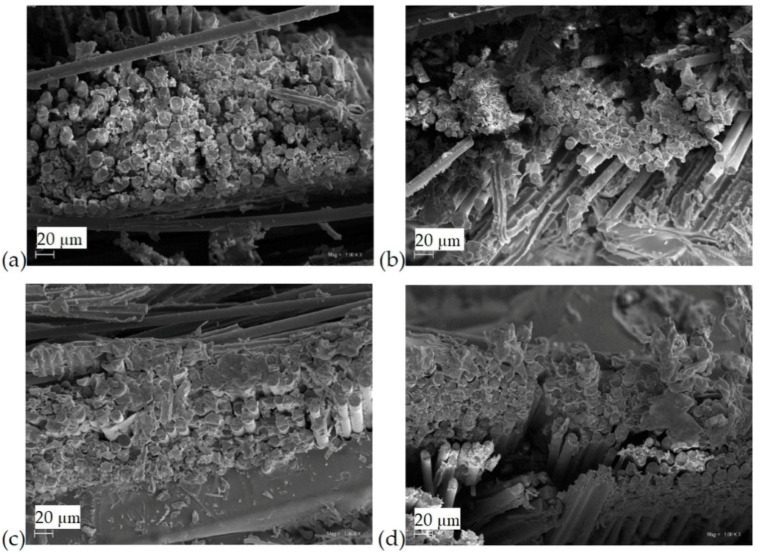
Structure of composites (**a**) 0PVC; (**b**) 2.5PVC; (**c**) 5PVC; (**d**) 10PVC.

**Figure 2:**
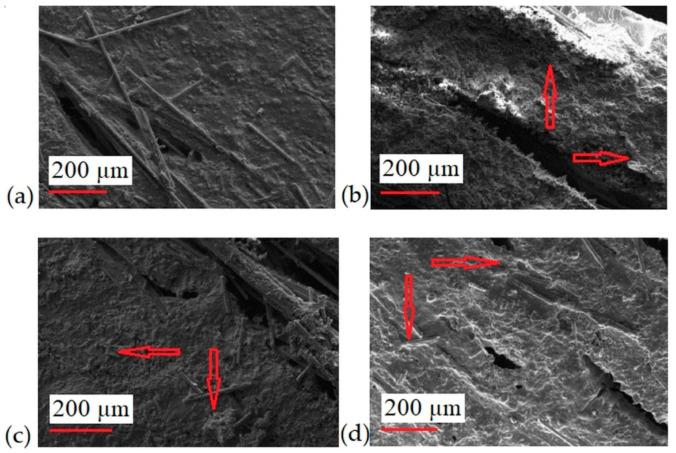
Structure of composites after ILSS test (**a**) 0PVC; (**b**) 2.5PVC; (**c**) 5PVC; (**d**) 10PVC.

The fracture morphology of composite samples was assessed using the SEM method. For all the tested samples, no significant differences were observed at the epoxy fiber-matrix interface (Figure 1a–d). This may indicate a good combination of all components in this layered composite. The fibers in the composites 5PVC and 10PVC were found to be more closely connected than in the reference sample (Figure 1c,d). Obtaining a favorable composite structure by properly connecting the epoxy matrix with the fiber and filler led to the improvement of the mechanical properties of the materials [29]. Moreover, SEM images of samples after ILSS tests are presented in Figure 2. The resulting pattern of damage in all cases was similar. The single pulled out fibers were not observed, which confirms their good connection with the matrix. Arrows indicate the distribution of PVC particles.

### 3.2. Thermomechanical Properties

**Figure 3:**
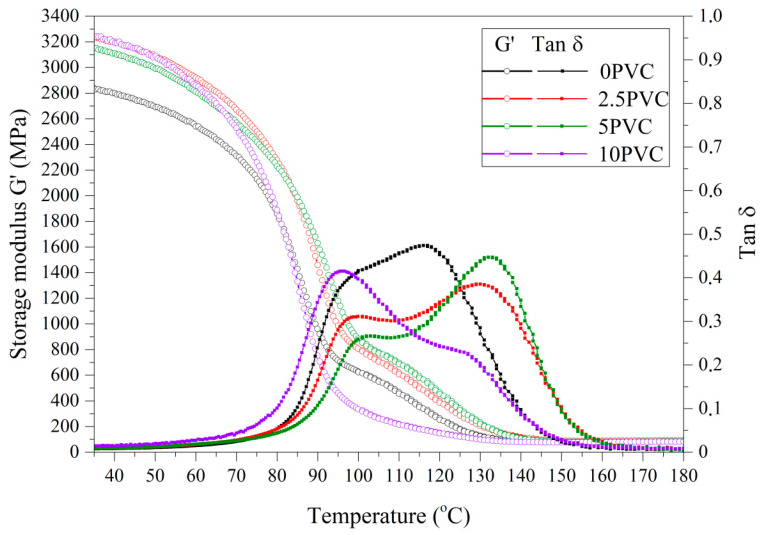
DMTA curves of investigated materials.

The thermomechanical properties of the composites are one of the critical criteria determining the area of their application as construction materials. Therefore, the dynamic thermomechanical analysis provides important information on material properties, such as glass transition temperature, storage modulus, and damping factor. The storage modulus describes the amount of energy stored by the sample during one cycle of measurement [30]. Figure 3 shows the dependence of storage modulus and damping factor on temperature. Two peaks were observed on the tan *δ* curves for composites with PVC; the first one in the temperature range of 90–100 °C, which can be attributed to the glass transition of poly(vinyl chloride), and the second one in the range of 120–130 °C, which can be attributed to the glass transition of epoxy resin. It should be emphasized that, for all the tested PVC-modified composites, the value of storage modulus was higher than for the reference sample. G’ values determined at 30 °C for 2.5PVC, 5PVC and 10PVC samples were similar and averaged 3200 MPa, whereas for the sample 0PVC it was 2870 MPa (Table 1). It can be concluded that well-dispersed PVC particles can improve stiffness and act as a reinforcement in epoxy composites, which is consistent with our previous work [28]. Thermomechanical properties of fiber-reinforced materials are strictly dependent on the type of fiber, its weave, the type of matrix used, its continuity and the presence of other reinforcing molecules. Mineral fillers, if not functionalized, most often physically interact with the polymer matrix, while PVC, due to the Cl atom present, may be reactive with non-crosslinked epoxy monomers [31]. Therefore, when analyzing the G’ value above the glass transition temperature at 130 °C, a favorable effect is observed with the presence of modifier particles in the epoxy matrix, because these values are much higher than for PVC for samples 2.5PVC and 5PVC. This may indicate the favorable thermomechanical properties of these materials and higher resistance to temperature changes [9].

### 3.3. Flexural Behaviors

**Figure 4:**
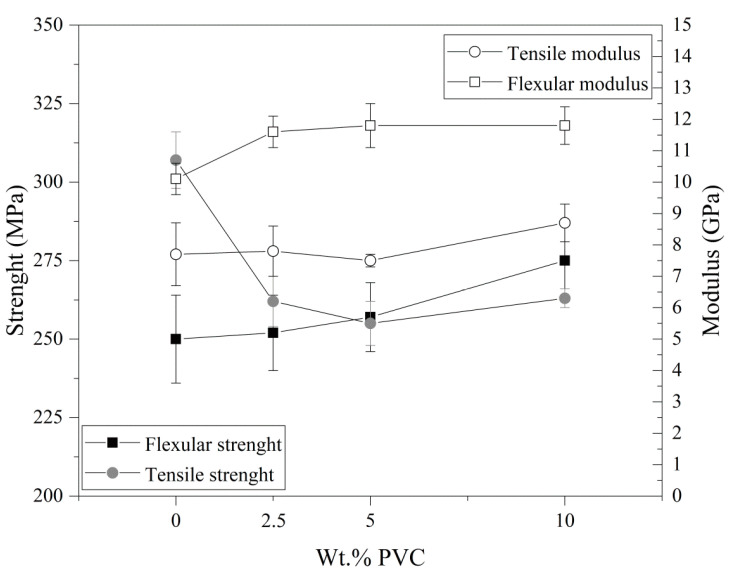
Mechanical properties of the composites.

The bending resistance of fiber-reinforced composite materials is important for their functional properties. Flexural strength values for the modified 2.5PVC and 5PVC composites were similar to the unmodified sample (Table 2). Figure 4 shows the dependence of the obtained results of mechanical tests on the content of the PVC additive. However, a significant improvement of this value is observed from 250 MPa to 275 MPa for the composite with the highest content of PVC powder (10 wt.%). The values of the flexural modulus were similar for all the modified materials and slightly higher than for the reference sample. The addition of PVC powder may improve the rigidity of the tested materials and make it possible to obtain a composite with specific functionality [21].

### 3.4. Tensile Behaviors

Tensile strength values usually differ from flexural strength. For the tested materials, a decrease in tensile strength was noted in the group of modified PVC composites compared to the unmodified composites (Table 2). Nevertheless, Young’s modulus values for 2.5PVC and 5PVC samples were similar to the 0PVC sample, and a significant improvement in this parameter was noted for the 10PVC sample. This is consistent with our previous results, where the addition of PVC powder improves the stiffness of materials [28]. In order to compare the obtained mechanical properties of the tested composites, a table with literature data on similar systems based on epoxy resin reinforced with basalt fiber and modified with powder fillers is presented (Table 3). In the case of 10-layer basalt–epoxy composites modified with graphene nanoplatelets (GNPs) (0.1–0.3 wt.%), similar level values of flexural strength (275 MPa) and tensile strength (263 MPa) were obtained for composites with PVC [22]. Langovan et al. described that the addition of silica nanoparticles (SiO_2_) at 1.5 wt.% to epoxy/basalt composite pipes improved their flexural properties [32]. In the case of nanofillers, their addition at the level of 0.1–1 wt.% is enough to obtain improved properties. For fillers with a larger particle size, a much larger amount of additive is required. The beneficial effect of introducing sawdust (at 2 wt.%) into the epoxy matrix for six-layer basalt composites was described by Surana et al. [27].

### 3.5. Interlaminar Shear Strength

**Figure 5:**
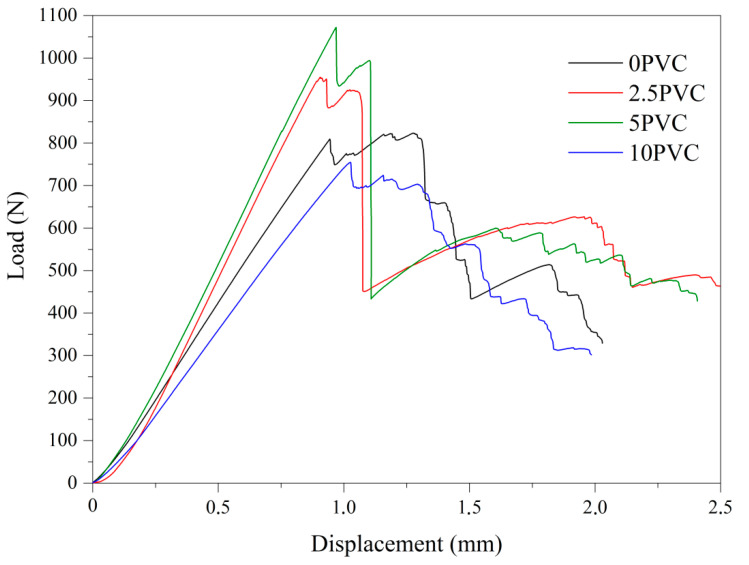
The curves of load on displacement for investigated materials.

Interlaminar Shear Strength is the possibility to resist the inter-laminar deformation under load [33]. This is especially important in the case of composites reinforced with fibers and modified with other particles. The composites ILSS as well as the maximum load values determined, and the maximum load are summarized in the Table 4. The ILSS values for the modified composites were higher than those of the reference sample, which may indicate the beneficial effect of the PVC addition, and is in good agreement with the results obtained in the flexural test. This confirms the good strength of the interfacial bond between all composite components. In the work of Scalici et al. [13] the ILSS value for eight-layer basalt–epoxy composites was 18.9 MPa for composites produced by resin infusion and 21.1 MPa for composites produced by vacuum bagging. Therefore, it can be concluded that the results obtained for the tested PVC composites are satisfactory. Moreover, according to the literature, the Cl atom of PVC can attack the oxirane ring and chemically bond to the epoxy matrix [31]. Additionally, the recorded maximum force for all PVC composites was higher than for the neat sample. The highest values ILSS and p were recorded for the 5PVC sample. In the case of 10PVC these values were lower, which may be due to the tendency of a large amount of PVC particles to agglomerate. Figure 5 presents the dependence of load on displacement for investigated materials. The curves of all samples are characterized by the first linear region, followed by a sudden decrease and increase in load.

### 3.6. Hardness

The Barcol hardness methods determine the hardness of composite materials by the depth of penetration of the indenter into the material [34]. Barcol hardness values were, respectively, 45 for the 0PVC sample, and an average of 55 for samples modified with powdered PVC, which confirmed the beneficial effect of introducing this additive into the epoxy matrix.

### 3.7. Thermal Stability

**Figure 6:**
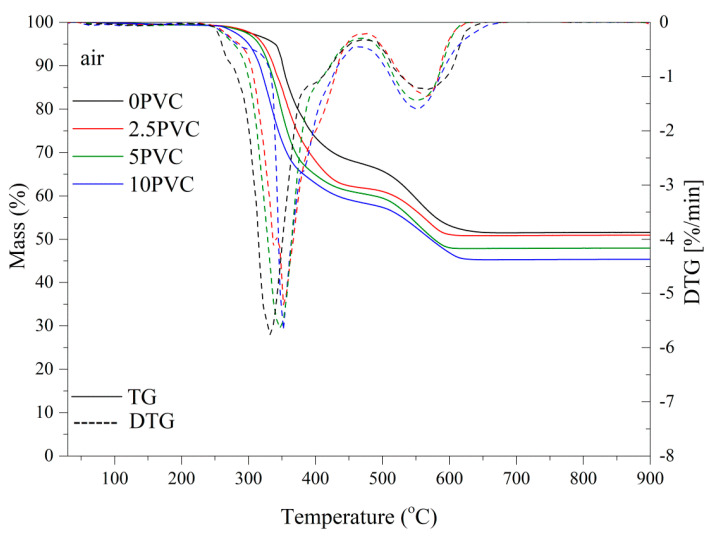
The characteristic thermogravimetric curves of the composites.

The thermogravimetric method was used to evaluate the thermal stability of the composites. The results are summarized in Table 5. The introduction of PVC powder reduced the T10% value of composites, caused by a partial distribution of PVC grains at a lower temperature than the pure epoxy resin. The residue mass after the test was similar in all cases, and on average reached 50 wt.%. It should be emphasized that the addition of PVC to the epoxy matrix delayed its degradation. This confirms the shift in the maximum decomposition temperature DTG peak towards higher temperatures for composite samples compared to the reference sample. The characteristic thermogravimetric curves are shown in Figure 6. A two-stage distribution was observed for all the tested composites.

### 3.8. Fire Behavior

Cone calorimeter tests were conducted to investigate the fire behavior of epoxy resin composites, and the relevant data, such as time to ignition (TTI), time to flameout (TTF), average heat release rate (av-HRR), total heat release (THR), maximum average rate of heat emission (MAHRE), fire residue and specific extinction area (SEA) are listed in Table 6.

As presented in Table 6, the addition of PVC powder resulted in TTI reduction, with the single exception of the 10PVC composite; moreover, in most cases, a higher TTF was observed.

**Figure 7:**
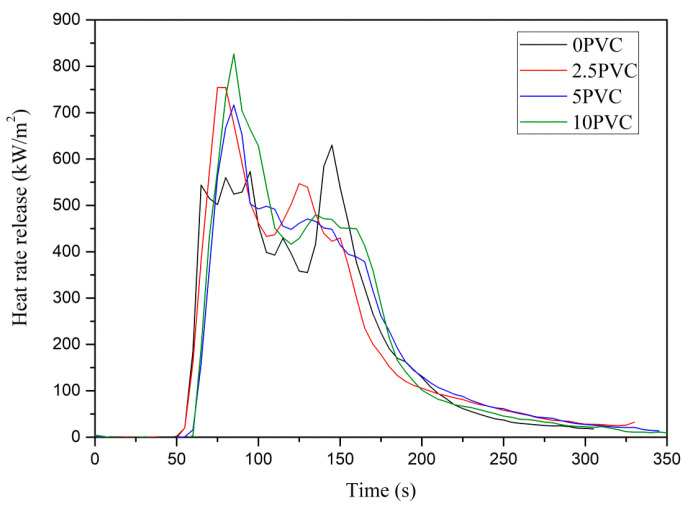
Representative heat release rate curves of the composites.

The HRR curves of composites are shown in Figure 7. All the investigated materials exhibited curves consisting of two maxima, the first peaks were a result of the rapid initial increase in HRR, while the second appeared at the end of burning. Contrary to composites without PVC, higher values were obtained for the first peak, and the second peak flattened as the proportion of the additive increased. The average values of HRR were similar to those obtained for the reference material, and a slight decrease was noted for composites with the highest amount of PVC only (Table 6). The congruous reduction in av-HRR values (by approx. 10%) for epoxy composites containing 10 wt.% of PVC powder was observed in our previous work [28]. PVC has self-extinguishing properties and does not self-ignite [35]. Howeever, the introduction of only high content of powdered PVC into the epoxy resin has a positive effect on the fire resistance of composites, such as a reduction in heat release rate. In our previous article, a reduction in HRR and increase in TTI was achieved with 40 wt.% of PVC additive [28].

Maximum average rate of heat emission (MAHRE), corresponding to the peak of the cumulative heat emission divided by time, is another important parameter, used to assess the influence of materials on fire development. Compared to the reference material, the MARHE of composites with PVC was higher and reached similar values regardless of the poly(vinyl chloride) contribution. As shown in Table 6, the total heat release (THR) of the 0PVC sample was 63 MJ/m^2^, while that of the composites, in most cases, increased due to the addition of PVC. In some cases THR may be dependent on a length of flame burning time (TTF), however, the 2.5PVC and 5 PVC samples showed similar values of TTF to the reference sample.

**Figure 8:**
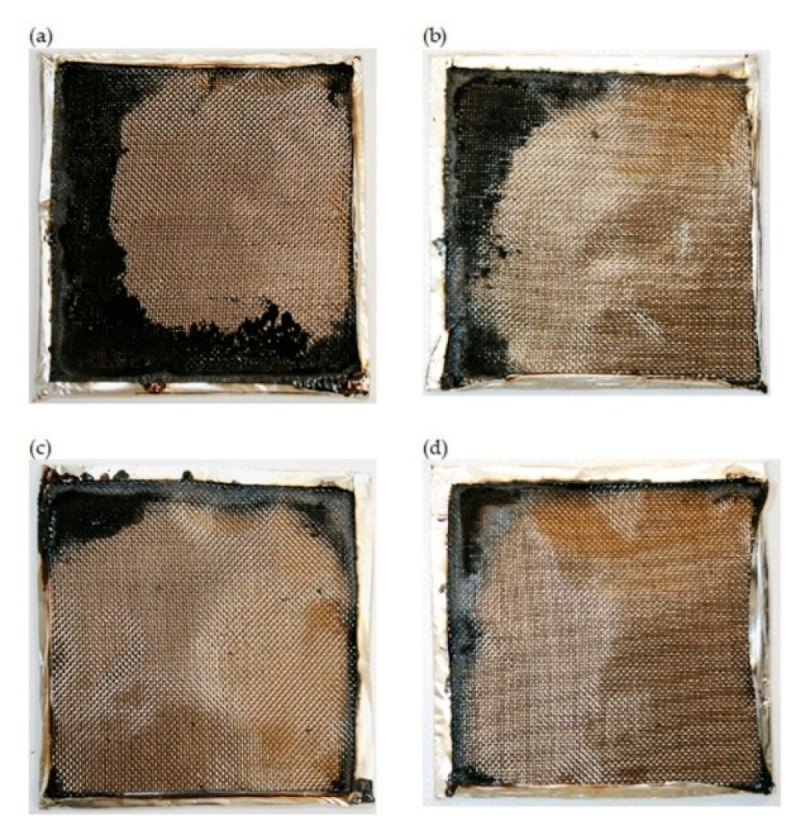
Photographs of samples after cone calorimetry tests: (**a**) 0PVC, (**b**) 2.5PVC, (**c**) 5PVC, (**d**) 10PVC.

Fire residues of the reference material and composites with 2.5–5 wt.% of PVC were similar and reached approx. 51%. The addition of a higher amount of thermoplastic powder caused a decrease in the investigated features. However, the results did not reflect the initial share of PVC. The reduction in the yield of residue with increasing PVC amount is confirmed by the photographs of samples after cone calorimetry tests (Figure 8). The addition of PVC powder did not have a positive effect on the smoke emission either. The specific extinction area (SEA) increased along with the amount of PVC, and the highest values (increase of approx. 7%) was recorded for composites modified with 10 wt.% of poly(vinyl chloride). The increase in this value was significant for the 10PVC sample, while for the 2.5PVC and 5PVC samples, these values were similar to the reference sample. The growth in the fumes emission as a result of PVC powder addition is consistent with the previous results [28]. 

## 4. Conclusions

The beneficial effect of introducing a powder filler in the form of poly(vinyl chloride) as a modifier in epoxy composites has been shown. The introduction of micron PVC powder into the epoxy matrix improved the thermomechanical properties of composites, such as storage modulus and mechanical properties, such as flexural strength and modulus and ILSS, as well as hardness. The TGA analysis has proven that the addition of PVC to the epoxy matrix retarded its rate of degradation. A slight decrease in the heat release rate was recorded for composites with the highest amount of PVC. Composites with 2.5 and 5 wt.% PVC showed similar fire properties to the reference sample. As for utility considerations, it is important to design materials that are safe for the environment. The introduction of powder fillers into layered composites is an easy method of obtaining materials with specific properties already at the stage of their components without the need for subsequent processing.

## Figures and Tables

**Table 1:** DMTA analysis results.

Name	G’(MPa) at 30 °C	G’(MPa) at 130 °C	Tg_I peak_ (°C)	Tg_II peak_ (°C)
0PVC	2870	128	116	−
2.5PVC	3250	225	97	130
5PVC	3180	221	100	132
10PVC	3260	100	96	127

**Table 2:** Mechanical properties of composites.

Name	Flexural Strength (MPa)	Flexural Modulus (GPa)	Tensile Strength (MPa)	Tensile Modulus (GPa)	Elongation at Break (%)
0PVC	250 ± 14	10.1 ± 0.5	307 ± 9	7.7 ± 1.2	4.7 ± 0.2
2.5PVC	252 ± 12	11.6 ± 0.5	262 ± 8	7.8 ± 0.8	4.4 ± 0.2
5PVC	257 ± 11	11.8 ± 0.7	255 ± 7	7.5 ± 0.2	4.1 ± 0.2
10PVC	275 ± 13	11.8 ± 0.6	263 ± 3	8.7 ± 0.6	4.4 ± 0.1

**Table 3:** Mechanical properties of basalt/epoxy composites with different filler.

wt.% of Fillers and Name	Flexural Strength (MPa)	Flexural Modulus (GPa)	Tensile Strength (MPa)	Tensile Modulus (GPa)	Ref.
0 GNPs	210	4.8	212	13.1	[18]
0.1 GNPs	273	8.1	240	15.9	
0.2 GNPs	232	7.8	224	14.2	
0.3 GNPs	220	5.7	223	13.3	
0 SiO_2_	108	9	−	−	
0.5 SiO_2_	109	9.1	−	−	[26]
1 SiO_2_	132	9.6	−	−	
1.5 SiO_2_	137	10.9	−	−	
0	−	−	194	7.2	
2 sawdust	−	−	252	7.5	[22]
4 sawdust	−	−	205	7.0	
6 sawdust	−	−	177	6.5	

**Table 4:** ILSS of composites.

Name	ILSS (MPa)	Max. Load, P (N)
0PVC	22.6 ± 0.6	756 ± 45
2.5PVC	23.9 ± 0.3	960 ± 25
5PVC	26.7 ± 0.4	1100 ± 22
10PVC	23.8 ± 0.8	806 ± 60

**Table 5:** TG and DTG values of the materials examined in air atmosphere.

Name	T10% (°C)	Residual Mass (%)	DTG Peak Temperature (°C)	Max Degradation Rate (%/min)
PVC	271.0	1.05	287.8	17.74
0PVC	350.4	51.5	331.6	5.78
2.5PVC	337.6	50.9	352.5	5.24
5PVC	330.7	47.9	347.2	5.64
10PVC	317.5	45.4	351.2	5.70

**Table 6:** Cone calorimeter results of investigated composites (irradiance 35 kW/m^2^).

Sample	TTI (s)	TTF (s)	av-HRR (kW/m^2^)	MARHE (kW/m^2^)	THR (MJ/m^2^)	Fire Residue (%)	SEA (m^2^/kg)
0PVC	63 (11 ^a^)	321 (32)	239.0 (8)	292.2 (10)	63.0 (2)	51.1 (3)	865.2 (32)
2.5PVC	61 (10)	333 (13)	242.0 (13)	318.1 (2)	66.2 (6)	50.5 (2)	848.3 (42)
5PVC	54 (12)	303 (25)	245.4 (34)	316.3 (18)	52.9 (20)	50.5 (1)	898.2 (28)
10PVC	63 (5)	375 (27)	215.1 (23)	315.2 (16)	68.1 (8)	47.5 (2)	923.7 (54)

^a^ The values in parentheses are the standard deviations.
